# Association of bone turnover markers and cognitive function in Chinese chronic schizophrenia patients with or without vitamin D insufficiency

**DOI:** 10.1186/s12888-023-05375-7

**Published:** 2023-11-22

**Authors:** Chen Ling, Liling Sun, Bei Luo, Haiyun Yu, Wei Li, Yating Yang, Huanzhong Liu

**Affiliations:** 1 Department of Psychiatry, School of Mental Health and Psychological Sciences, Anhui Medical University, Hefei, 230032 China; 2 Department of Psychiatry, Chaohu Hospital of Anhui Medical University, Hefei, 238000 China; 3 Department of Psychiatry, Anhui Psychiatric Center, Anhui Medical University, Hefei, 230032 China

**Keywords:** Bone turnover markers, Cognitive function, Vitamin D, Schizophrenia

## Abstract

**Background:**

Increasing evidence shows that bone turnover markers (BTMs) and vitamin D can affect human cognitive function. However, there are few studies that have investigated the association between BTMs and cognitive function in chronic schizophrenia patients. The aim of this study was to investigate the relationship between BTMs and cognitive function in chronic schizophrenia patients with or without vitamin D insufficiency (VDI).

**Methods:**

In all, 118 chronic schizophrenia patients were enrolled in this cross-sectional study. Repeatable Battery for the Assessment of Neuropsychological Status (RBANS) was applied to evaluate the cognitive function of the subjects. Blood analysis included bone turnover markers, vitamin D levels, and glycolipid levels.

**Results:**

Relative to 72 vitamin D-sufficient (VDS) patients, 46 VDI patients had higher bone resorption markers levels and lower bone formation markers levels. Regression analysis showed that, in the total sample, CTX and language function exhibited independent positive correlation (*p* = 0.027, R^2^ change = 0.042), and in the VDS group, procollagen type I N-terminal propeptide (PINP) was independently negatively correlated with language function (*p* = 0.031, R^2^ change = 0.065), while the positive correlation between osteopontin (OPN) and delayed memory remained in the VDI group (*p* = 0.036, R^2^ change = 0.083).

**Conclusion:**

Our study showed an association between the levels of BTMs and cognitive function among chronic schizophrenia patients. This correlation may have different mechanisms of action at different vitamin D levels.

## Introduction

Schizophrenia is considered to be one of the most serious of all psychiatric illnesses [1], and is one of the top 10 reasons for disability globally [2]. According to the most recent epidemiological survey in Chinese, the weighted lifetime prevalence of schizophrenia is 0.7% [3]. Chronic schizophrenia patients experience various forms of cognitive impairment. These impairments negatively affect daily functional outcomes and contribute to the disabling nature of schizophrenia [4]. Studies have demonstrated that functional impairment is closest correlated with cognitive impairment rather than with the severity of psychiatric symptoms [5]. Therefore, it is important to explore the potential influencing factors of cognitive function to improve the lives of patients with schizophrenia.

In addition to being a structural organ, bones are also metabolically active endocrine organs. Many experimental and clinical studies have found that bone has crosstalk with the brain through bone-related peptides secreted by itself [6]. For example, osteocalcin (OCN), which is involved in bone formation, has been proven to be able to cross the blood-brain barrier (BBB) and has important effects on brain development, learning and memory in mice [7]. A study of healthy individuals found that plasma levels of OCN were positively related to executive function and global cognitive performance in older women. [8]. Osteopontin (OPN), which is involved in bone resorption, also crosses the BBB and has conflicting effects in the brain. OPN exerts neuroprotection or deleterious neuroinflammation through the activation of different signaling pathways [9]. In a study of patients with schizophrenia, the level of OPN was found to be significantly correlated with patients’ positive and negative syndrome scale (PANSS) general and total scores [10]. Collectively, these findings suggest that the bone can have an impact on the brain through its endocrine functions and affect human cognitive function.

Bone turnover markers (BTMs) can more subtlety reflect changes in bone metabolism than bone mineral density (BMD). We can hypothesize that BTMs are more suitable for detecting bone resorption or bone formation in schizophrenia patients [11]. For instance, one study found elevated bone formation and bone resorption markers in premenopausal and regularly menstruating women with schizophrenia, while the spine and hip BMD was within normal range [12]. International clinical guidelines recommend two BTMs: C-terminal cross-linking telopeptide of type I collagen (CTX) and procollagen type I N-terminal propeptide (PINP), respectively, as markers of bone resorption and bone formation [13].

Vitamin D affects bone metabolism through direct its effects on bone as well as its effects on intestinal calcium absorption and parathyroid hormone (PTH) levels. A study in healthy Chinese populations has shown that supplementation of vitamin D has different effects on various BTMs [14]. Vitamin D mediates multiple biological targets, including neurons and glial cells, through the vitamin D receptor (VDR) [15]. This vitamin is involved in the regulation of neurotransmission, neuroprotection, and neuroimmune regulation, such as the control of calcium homeostasis in the neurons of the hippocampus [16]. In a study of older adults living in the community, higher brain vitamin D concentrations reduced the odds of dementia or mild cognitive impairment (MCI) by 25–33% [17]. And in patients with schizophrenia, vitamin D deficiency was significantly correlated with a decrease in processing speed and verbal memory [18]. Furthermore, there was a double-blind, randomized controlled study that confirmed that supplementation of vitamin D tended to improve cognition in chronic schizophrenia patients with low initial vitamin D levels [19]. Based on the American Endocrine Society clinical practice guideline, we can define 25-hydroxy-vitamin D (25-OH-D) below 30 ng/ml as vitamin D insufficiency (VDI) and 25-OH-D above 30 ng/ml as vitamin D sufficiency (VDS) [20].

Many previous studies have examined the factors influencing BTMs in schizophrenia patients, but there are few studies linking the levels of BTMs and cognitive function in schizophrenia patients. In this study, four markers of bone metabolism were selected, representing the processes of bone resorption and bone formation, respectively, and bone metabolism and cognitive function and their correlation were explored under different vitamin D levels.

## Methods

### Subjects

We enrolled 118 inpatients with chronic schizophrenia from the Chaohu Hospital of Anhui Medical University. All subjects matched the following criteria for inclusion: (1) consistent with the diagnosis of schizophrenia by two clinical psychiatrists according to the fifth edition of the Diagnostic and Statistical Manual of Mental Disorders (DSM-5); (2) Han Chinese, 18 to 60 years of age, education years ≥ 5 ; (3) had at least three years of disease duration, and received a fixed-dose of antipsychotic medications in at least 12 months prior to being recruited [21]; (4) were able to understand and sign the informed consent; (5) had no known history of alcohol dependence or drug abuse.

Subjects who meet the following criteria have been excluded: (1) met the DSM-5 diagnostic criteria for serious psychiatric disorders other than schizophrenia; (2) had serious physical diseases such as heart disease, cerebral infarction, and chronic liver or kidney disease; (3) had known endocrine disorders that affect bone metabolism, like diabetes or thyroid disorders [11]; (4) had used drugs that clearly affect bone metabolism or vitamin supplements in addition to antipsychotic drugs within three months; (5) pregnant or lactating women.

This study had the approval of the Ethics Committee of Chaohu Hospital, affiliated with Anhui Medical University (Approval No. kyxm-202211-002), with all participants signed informed consent.

### Clinical and Cognitive Assessment

All subjects completed an interview. General demographics and clinical characteristics of all participants were collected by the researchers. PANSS was used by two trained psychiatrists to assess psychiatric symptoms in schizophrenia patients. Antipsychotic treatment doses were converted to chlorpromazine equivalent doses through the internationally recognized defined daily doses (DDDs) method [22].

We used the Chinese version of Repeatable Battery for the Assessment of Neuropsychological Status (RBANS) for measuring the cognitive function of patients. The scale consists of five indicators: immediate memory, visuospatial/constructional, language, attention, and delayed memory [23]. The scale has shown adequate validity and repeat measurement reliability in Chinese populations [24].

### Biochemical assays

All subjects were collected 20ml of fasting venous blood from 6:00 am to 8:00 am on the day of the interview assessment. Fasting blood glucose (FBG), triglyceride (TG), total cholesterol (TC), low-density lipoprotein (LDL), and high-density lipoprotein (HDL) were measured. FBG was measured by the oxidase method. TG was determined by the TG_2 reagent method. The Cholesterol_2 Reagents method was used to detect TC. LDL was measured using the LDL Cholesterol Direct method. HDL was measured using the Direct HDL Cholesterol Reagents method. We used 10 ml of blood samples to detect BTMs and vitamin D levels. The plasma separated by centrifugation is stored in a -80 °C refrigerator until the time of the assay. We detected CTX, PINP, OCN, OPN and 25-OH-D by enzyme-linked immunosorbent assay (ELISA) method on a Rayto RT-6100 microplate analyzer.

### Statistical analysis

We analyzed the data using SPSS23.0 software. Continuous variables are presented as mean ± standard deviation, and categorical variables are presented as percentages. First, we divided the subjects into two groups based on vitamin D levels: VDS (25-OH-D>30ng/ml ) and VDI (25-OH-D ≤ 30ng/ml). Firstly, we analyzed the general demographic characteristics and clinical data between the two groups. The continuous variables that conformed to the normal distribution were compared by *t*-test, the non-normally distributed continuous variables were compared by Mann-Whitney U-test, and the categorical variables were tested by chi-square test. BTMs and RBANS scores were also compared according to the above method.

Then, in the total sample and in each subgroup, Spearman correlations analysis was performed between BTMs and cognitive function. Multivariate stepwise regression analysis was performed with the RBANS scores for each domain and the total score as dependent variables to further determine the relationship between BTMs and cognition in the VDS, VDI group and total sample, and some potential confounders were included in the analysis, such as age, gender, years of education, BMI, PANSS score, drug equivalents, FBG, blood lipids and whether the patient is taking benzodiazepines and Benzhexol.

## Results

### Demographics and clinical features

In total, 118 subjects completed the entire study process. Among them, VDS has 72, and VDI has 46. Table 1 shows that there were no significant differences between VDS and VDI in terms as to age, sex, years of education, BMI, drug equivalent, and disease duration. The PANSS total score, FBG, TG, TC and LDL of VDI were slightly higher than that of VDS, and the high-density lipoprotein was slightly lower than that of VDS, but there was no statistical significance.

**Table 1:** Demographics and clinical features of schizophrenia patients with or without vitamin D insufficiency

	Total sample (n = 118)	VDS (n = 72)	VDI (n = 46)	t/Z/ X^2^	*P*
Age (years)	43.13 ± 10.17	42.51 ± 10.08	44.09 ± 10.34	-0.819	0.415
Female (%)	46 (38.98%)	31 (43.06%)	15 (32.61%)	1.288	0.256
Education (years)	9.56 ± 2.91	9.65 ± 2.97	9.41 ± 2.83	-0.4	0.689
BMI (kg/m^2^)	24.58 ± 3.80	24.37 ± 3.68	24.90 ± 4.00	-0.728	0.468
Antipsychotic dose (mg/day)	733.82 ± 318.95	761.50 ± 319.77	690.49 ± 316.25	-1.4	0.161
Using Benzhexol	63 (53.39%)	40 (55.56%)	23 (50.00%)	0.348	0.555
Using benzodiazepines	6 (5.08%)	3 (4.17%)	3 (6.52%)	——	0.677^a^
Duration of illness (years) PANSS	17.19 ± 9.75	16.88 ± 10.00	17.67 ± 9.45	-0.4	0.689
Positive symptom	14.39 ± 4.85	14.29 ± 4.02	15.48 ± 4.71	-1.459	0.145
Negative symptom	16.89 ± 6.24	17.32 ± 5.84	17.33 ± 5.53	-0.058	0.954
General psychopathology	32.29 ± 6.36	31.56 ± 5.77	33.44 ± 7.09	-1.213	0.225
Total score	64.36 ± 13.16	63.17 ± 12.01	66.24 ± 14.73	-1.065	0.225
FBG (mmol/l)	5.20 ± 1.22	5.06 ± 0.74	5.42 ± 1.71	-0.757	0.449
TC (mmol/l)	4.46 ± 0.94	4.38 ± 0.92	4.58 ± 0.97	-1.3	0.194
TG (mmol/l)	1.76 ± 1.22	1.60 ± 0.75	2.00 ± 1.69	-0.767	0.443
LDL (mmol/l)	2.60 ± 0.81	2.57 ± 0.80	2.70 ± 0.90	-0.788	0.432
HDL (mmol/l)	0.95 ± 0.20	0.96 ± 0.21	0.93 ± 0.18	0.731	0.466

VDS: Vitamin D Sufficiency; VDI: Vitamin D Insufficiency; PANSS: Positive and Negative Syndrome Scale; BMI: Body Mass Index; FBG fasting blood glucose, TG triglyceride, TC total cholesterol, HDL high-density lipoprotein, LDL low-density lipoprotein. Bolded P values < 0.05

a: 2 cells have expected count less than 5. So only Fisher’s exact test results are shown

### Cognition and bone turnover markers

In Table 2, compared with VDS group, the bone formation index in VDI group was significantly lower, with a statistically significant difference in OCN (*t* = 2.74, *p* = 0.007), while the bone resorption index was higher in the VDI group, with a statistically significant difference in CTX (*t* = -3.49, *p* = 0.001). But there was no significant difference in cognitive function between the VDS and VDI groups.

**Table 2:** Cognition and bone turnover markers of schizophrenia patients with or without vitamin D insufficiency

	Total sample (n = 118)	VDS (n = 72)	VDI (n = 46)	t/Z	*P*
RBANS					
Immediate Memory	61.08 ± 19.53	60.56 ± 18.91	61.89 ± 20.66	-0.166	0.868
Visuospatial	79.42 ± 17.19	79.38 ± 17.49	79.48 ± 16.91	-0.263	0.793
Language	79.46 ± 14.66	79.32 ± 14.94	79.67 ± 14.38	-0.053	0.958
Attention	87.88 ± 12.83	88.04 ± 12.69	87.63 ± 13.17	-0.048	0.962
Delayed Memory	68.78 ± 20.72	67.89 ± 20.58	70.17 ± 21.10	-0.744	0.457
Total score	68.97 ± 14.59	68.53 ± 14.14	69.65 ± 15.41	-0.271	0.787
CTX (pg/ml)	330.25 ± 60.82	315.33 ± 59.88	353.61 ± 55.19	-3.49	0.001
PINP (ng/ml)	74.69 ± 14.94	76.80 ± 15.20	71.39 ± 14.04	1.943	0.054
OCN (ng/ml)	58.29 ± 14.42	61.12 ± 13.43	53.86 ± 14.94	2.74	0.007
OPN (ng/ml)	42.09 ± 9.99	40.84 ± 9.61	44.04 ± 10.35	-1.711	0.090

RBANS: Repeatable Battery for the Assessment of Neuropsychological Status; CTX: C-terminal cross-linking Telopeptide of type I collagen; PINP: Procollagen type I N-terminal Propeptide; OCN: Osteocalcin; OPN: Osteopontin. Bolded P values < 0.05

### Correlation between cognitive function and bone turnover markers

Due to the significant differences in BTMs between VDS and VDI groups, we analyzed the correlation between BTMs and cognitive function in the two groups and total sample. Table 3 shows that in the total sample, CTX and language function showed a positive correlation (*r* = 0.229, *p* < 0.05), while PINP was negatively correlated with language function (*r* = -0.189, *p* < 0.05). This correlation is consistent with the VDS group (all *p* < 0.05), whereas only in the VDS group, PINP and RBANS total scores showed a negative correlation (*r* = -0.240, *p* < 0.05). In the VDI group, PINP was positively associated with delayed memory (*r* = 0.320, *p* < 0.05), in contrast to OPN, which was negatively associated with delayed memory (*r* = -0.330, *p* < 0.05). In both groups and total sample, there was no significant correlation to be found between OCN and any cognitive function variable.

**Table 3:** Correlation between cognitive function and bone turnover markers

	CTX	PINP	OCN	OPN
Total sample				
Immediate Memory	0.067	-0.052	0.045	0.053
Visuospatial	-0.046	0.059	-0.001	-0.058
Language	0.229*	-0.189*	-0.087	0.135
Attention	-0.054	-0.024	0.048	-0.061
Delayed Memory	0.014	0.009	0.085	-0.068
Total score	0.040	-0.061	0.031	0.006
VDS				
Immediate Memory	0.107	-0.176	0.012	0.159
Visuospatial	0.066	-0.003	-0.064	0.031
Language	0.266*	-0.273*	-0.125	0.196
Attention	-0.055	-0.069	0.036	0.015
Delayed Memory	0.101	-0.172	0.053	0.099
Total score	0.133	-0.240*	-0.024	0.160
VDI				
Immediate Memory	-0.024	0.136	0.120	-0.095
Visuospatial	-0.274	0.154	0.131	-0.259
Language	0.206	-0.093	-0.043	0.030
Attention	-0.068	0.055	0.096	-0.171
Delayed Memory	-0.203	0.320*	0.228	-0.330*
Total score	-0.146	0.175	0.147	-0.203

CTX: C-terminal cross-linking Telopeptide of type I collagen; PINP: Procollagen type I N-terminal Propeptide; OCN: Osteocalcin; OPN: Osteopontin. Data are shown as Spearman correlation coefficient (rho). *P < 0.05

### Multivariate stepwise regression analysis of bone turnover markers with cognitive function

In order to further clarify the correlation between the above BTMs and cognitive function, we performed stepwise multiple linear regression and corrected potential confounding factors (age, gender, years of education, BMI, PANSS score, drug equivalents, FBG, blood lipids and whether the patient is taking benzodiazepines and Benzhexol) in each group and the total sample. Table 4 shows that in the total sample, CTX and language function exhibited independent positive correlation (B = 0.050, *p* = 0.027, 95%CI = 0.006 ~ 0.093, R^2^ change = 0.042). In the VDS group, there was an independent negative correlation between PINP and language function (B = -0.250, *p* = 0.031, 95%CI = -0.477~-0.024, R^2^ change = 0.065), while in the VDI group, the positive correlation was still present between OPN and delayed memory. (B = -0.586, *p* = 0.036, 95%CI = -1.132~-0.041, R^2^ change = 0.083).

**Table 4:** Multivariate stepwise regression analysis of bone turnover markers with cognitive function

Relationships	Regression coefficient (B)	95%CI	R^2^ change	*P* value
*Total sample*				
CTX and Language	0.050	0.006 ~ 0.093	0.042	0.027
*VDS*				
PINP and Language	-0.250	-0.477~-0.024	0.065	0.031
*VDI*				
OPN and Delayed Memory	-0.586	-1.132~-0.041	0.083	0.036

## Discussion

Our current study shows that chronic schizophrenia patients consistent with vitamin D deficiency had poorer bone metabolism than vitamin D-sufficient patients, as evidenced by lower levels of bone formation markers and higher levels of bone resorption markers in the VDI group. We also found a correlation between BTMs and different domains of cognitive function, and interestingly, this correlation appeared to differ at different vitamin D levels. After adjustment for confounders, we found that OPN, which reflects bone resorption, was negatively correlated with delayed memory when vitamin D was deficient, while PINP, which measures bone formation, was negatively correlated with language function when patients had sufficient vitamin D levels. Corresponding to the correlation found when vitamin D levels were sufficient, CTX, which represents bone resorption, was positively correlated with language function in the total sample.

### Relationship between vitamin D levels and bone turnover markers

The significant impacts of vitamin D on bone metabolism are well established, and these effects are reflected in BTMs. To meet the calcium balance in the body, vitamin D and its metabolites enhance mineral deposits in the bone matrix when sufficient calcium is available, while in calcium deficiency, vitamin D promotes bone resorption while inhibiting bone mineralization [25]. This partly explains that in our study, patients with chronic schizophrenia who had vitamin D concentrations greater than 30 ng/ml had lower CTX and OPN levels but higher PINP and OCN levels. However, the results of various studies are inconsistent regarding the relationship between vitamin D concentrations and bone turnover marker levels. A large cross-sectional study of Chinese adults found that different vitamin D concentrations were negatively associated with bone metabolism markers, whether it was osteogenic or osteoclastic markers [26]. Another study, also conducted in Chinese adults, found that supplementation of vitamin D did not significantly affect CTX and PINP levels, while subjects with vitamin D concentrations above 30 ng/mL had increased CTX but no difference in PINP [14]. In contrast, it has been shown that in young postmenopausal women, improved vitamin D status can result in a significant decrease in serum PINP and CTX levels [27].

### The association between vitamin D levels and cognitive function

The association between vitamin D levels and cognitive function is inconsistent across studies. In healthy individuals, there may be an association between low vitamin D levels and cognitive function [15], and several RCT studies have shown that vitamin D supplementation positively affects cognitive function in people whose vitamin D levels were low at baseline [28–30]. However, in a cross-sectional study of bipolar patients, there was no relationship between cognitive function and vitamin D levels in bipolar patients [31]. Consistent with this finding, our findings showed no difference between subject RBANS scores for VDS and VDI in patients with chronic schizophrenia.

### Relationship between OPN and delayed memory in vitamin D insufficiency

Our study found that in chronic schizophrenic patients with vitamin D insufficiency, OPN concentrations were inversely correlated with delayed memory scores. OPN and vitamin D are not only closely related to the dynamic changes of bones but also can be used as markers of oxidative stress or inflammation [32]. The relationship between OPN and cognitive function has been verified in multiple studies. A study in a mouse model of AD found that the level of OPN-producing CD11c + microglia was strongly correlated with the extent of cognitive impairments and AD neuropathology [33]. In a cross-sectional survey of patients with cerebrovascular disease and neurodegenerative dementia, increased OPN was significantly related to vascular cognitive deficits and significantly related to neuroimaging markers of cerebrovascular disease and neurodegeneration [34]. One study found an association between OPN and schizophrenia and that OPN gene expression was upregulated in first-episode psychosis (FEP) patients [35]. Although current studies point to OPN affecting cognitive function by acting in oxidative stress-related pathways, Although the current study points to OPN affecting cognitive function by acting on oxidative stress-related pathways, the interaction between bone metabolism and OPN and, eventually, its impact on cognitive function also deserves attention.

### Relationship between PINP and language function when vitamin D is sufficient

Interestingly, our study found that in patients with sufficient vitamin D levels, PINP and language function were independently associated even after accounting for confounding factors, which was rarely reported in previous studies. And correspondingly, when vitamin D levels were not considered, CTX levels correlated with language function in the total sample. In an RCT of 103 community-dwelling older adults, no association was found between PINP and domains of cognitive function at baseline and after 18 months, whereas CTX, although associated with overall cognitive change at 18 months, was not correlated with any one specific cognitive domain and did not independently predict cognitive decline [36]. Although PINP cannot directly cross the BBB to affect the brain, the pathogenesis of schizophrenia appears to affect bone metabolism. The dopamine hypothesis is the most classic hypothesis on the pathogenesis of schizophrenia in the past forty years [37], and dopamine also affects bone metabolism. For example, dopamine inhibits osteoclast differentiation through D2 receptors [38], and activation of dopamine receptor D1 not only stimulates bone differentiation but also reduces bone loss [39], while PINP reflects the level of bone formation. Our research shows that there is a correlation between PINP and the cognitive function of patients with schizophrenia, which may be produced through the different effects of dopamine on various receptors, but the specific causal relationship still needs further study.

### No correlations were found between OCN and the various cognitive domains

It is worth mentioning that OCN has proved its correlation with cognitive function in many previous studies [8, 40], but it was not found in our study. As for why our results did not show the positive associations shown in previous studies, we speculate that the previous associations were found in more homogeneous populations, such as elderly women and obese patients. Results of an RCT of community-dwelling older adults support our negative findings [36].

### Potential threshold effects between vitamin D and BTMs

Importantly, our study found that the correlations between the levels of bone turnover markers and cognitive function appeared to be reversed at different vitamin D concentrations. This is possible because there is some threshold effect between vitamin D and BTMs, thus exhibiting different effects on cognition. In previous studies, different cut-off points of vitamin D levels could be used as markers of changes in BTMs. This potential threshold effect seems to result from the effect of vitamin D on PTH. In various previous studies, it was found that a rapid increase in PTH levels was observed when vitamin D concentrations were below 20 ng/ml [26], while PTH levels showed a steady state when vitamin D concentrations were above 30 ng/ml [41]. And in addition to the familiar effects of PTH on bone, there are many studies that have found a correlation between PTH and cognitive impairment. Possible mechanisms of action include PTH binding to receptors in the brain and inducing apoptosis through calcium overload [42]; or affecting cerebral blood flow through vasoactive effects, resulting in decreased cognitive function [43].

### Limitations

Our study has some limitations. Firstly, our current study presents only a cross-sectional result, and further longitudinal studies to clarify the causal relationship between bone metabolism and cognitive function are needed. Secondly, this study lacked a healthy control group to determine the differences in bone metabolism between schizophrenic and normal populations. Thirdly, our subjects are only inpatients. Although their diet and daily routine are relatively consistent—these are considered to have an impact on bone metabolism and vitamin D levels, the results may still be biased by the selection of samples. Finally, we could not rule out the effects of different drugs on skeletal or cognitive performance.

## Conclusion

We found significant differences in the levels of BTMs in chronic schizophrenia patients with different vitamin D levels, and these markers were correlated with cognitive function, and this correlation seemed to be the opposite, suggesting that there may be a threshold for vitamin D effect. OPN was negatively associated with delayed memory in patients with vitamin D levels below 30 ng/ml, whereas PINP was independently and negatively associated with language function in patients with vitamin D levels above 30 ng/ml. Positive correlation between CTX and language function in the total sample when vitamin D levels are not considered. Our study suggests that there may be some interaction between bone status and brain function in schizophrenia patients. To preserve cognitive function and restore social functioning in schizophrenia patients, further longitudinal studies are required to examine the interaction of bone metabolism and neuromodulation.

## Data Availability

As this study is still ongoing, the raw datasets for the current study will not be available until the end of this research project. The data used for this study are available from the corresponding author upon reasonable request.
